# Ekbom síndrome (Parasitosis delirium): Cocaine Use vs. Psychotic Depression. A case report

**DOI:** 10.1192/j.eurpsy.2024.851

**Published:** 2024-08-27

**Authors:** E. Arroyo Sánchez, J. Gimillo Bonaque, P. Setién Preciados, C. Díaz Mayoral

**Affiliations:** Hospital Universitario Príncipe de Asturias, Madrid, Spain

## Abstract

**Introduction:**

Parasitosis delirium represents a rare mono-symptomatic psychosis characterized by the delirious firm belief of the patient, against all evidence, of being infested by cutaneous parasites. The syndrome affects in particular middle-aged women, and can be the single manifestation of psychological uneasiness or represent one of the aspects of a more complex psychiatric case, compromising almost totally any normal daily work and/or social activity. It is often accompanied by a refusal to seek psychiatric care. This condition can be associated with various underlying causes, including substance use disorders and psychotic depression. Understanding the differences and similarities between delirium of infestation in the context of cocaine use and psychotic depression is crucial for accurate diagnosis and effective treatment.

**Objectives:**

This study aims to compare and contrast the clinical features, etiology, and treatment approaches of delirium of infestation in individuals with cocaine use and those with psychotic depression. By examining these two distinct populations, we can gain insights into the unique challenges and considerations associated with each condition.

**Methods:**

A case report of a 44-year-old woman with delirium of parasitosis, depressive symptoms and cocaine use in the last three days. Also a comprehensive literature review using the PubMed database to identify relevant clinical articles on delirium of infestation, cocaine use, and psychotic depression.

**Results:**

Cocaine use and psychotic depression can both cause delirium of infestation. Cocaine-induced delirium is characterized by agitation, paranoia, and delusions of infestation. Psychotic depression is characterized by a depressed mood, delusions, and hallucinations. Delusions of infestation are a common feature of bothconditions. However, the underlying mechanisms and treatment approaches differ. Cocaine-induced delirium is primarily associated with the acute effects of cocaine on the central nervous system, while psychotic depression involves a complex interplay of biological, psychological, and environmental factors. Treatment for cocaine-induced delirium involves addressing the underlying cocaine use, while treatment for psychotic depression involves antidepressant and antipsychotic medications. Otherwise, Anti-Parkinson drugs were most frequently associated with delusional infestation

**Conclusions:**

Delirium of infestation can occur in individuals with cocaine use and those with psychotic depression, albeit with different etiologies. Clinicians should consider the underlying cause when diagnosing and treating patients with this condition. Further research is needed to explore the specific neurobiological mechanisms and optimal treatment strategies for delirium of infestation in these distinct populations.

**Disclosure of Interest:**

None Declared

